# A Novel Environmental Azole Resistance Mutation in *Aspergillus fumigatus* and a Possible Role of Sexual Reproduction in Its Emergence

**DOI:** 10.1128/mBio.00791-17

**Published:** 2017-06-27

**Authors:** Jianhua Zhang, Eveline Snelders, Bas J. Zwaan, Sijmen E. Schoustra, Jacques F. Meis, Karin van Dijk, Ferry Hagen, Martha T. van der Beek, Greetje A. Kampinga, Jan Zoll, Willem J. G. Melchers, Paul E. Verweij, Alfons J. M. Debets

**Affiliations:** a Laboratory of Genetics, Wageningen University, Wageningen, The Netherlands; b Department of Medical Microbiology and Infectious Diseases, Canisius Wilhelmina Hospital (CWZ), Nijmegen, The Netherlands; c Centre of Expertise in Mycology Radboudumc/CWZ, Nijmegen, The Netherlands; d Department of Medical Microbiology, VU University Medical Centre, Amsterdam, The Netherlands; e Department of Medical Microbiology and Infection Control, Leiden University Medical Center, Leiden, The Netherlands; f Department of Medical Microbiology & Infection Prevention, University of Groningen, University Medical Center Groningen, Groningen, The Netherlands; g Department of Medical Microbiology, Radboud University Medical Centre, Nijmegen, The Netherlands; CDC

**Keywords:** *Aspergillus fumigatus*, ascospores, azole resistance, compost heap, conidiospores, hot spot for resistance development, novel mutation, sexual reproduction

## Abstract

This study investigated the dynamics of *Aspergillus fumigatus* azole-resistant phenotypes in two compost heaps with contrasting azole exposures: azole free and azole exposed. After heat shock, to which sexual but not asexual spores are highly resistant, the azole-free compost yielded 98% (49/50) wild-type and 2% (1/50) azole-resistant isolates, whereas the azole-containing compost yielded 9% (4/45) wild-type and 91% (41/45) resistant isolates. From the latter compost, 80% (36/45) of the isolates contained the TR_46_/Y121F/T289A genotype, 2% (1/45) harbored the TR_46_/Y121F/M172I/T289A/G448S genotype, and 9% (4/45) had a novel pan-triazole-resistant mutation (TR_46_^3^/Y121F/M172I/T289A/G448S) with a triple 46-bp promoter repeat. Subsequent screening of a representative set of clinical *A. fumigatus* isolates showed that the novel TR_46_^3^ mutant was already present in samples from three Dutch medical centers collected since 2012. Furthermore, a second new resistance mutation was found in this set that harbored four TR_46_ repeats. Importantly, in the laboratory, we recovered the TR_46_^3^ mutation from a sexual cross between two TR_46_ isolates from the same azole-containing compost, possibly through unequal crossing over between the double tandem repeats (TRs) during meiosis. This possible role of sexual reproduction in the emergence of the mutation was further implicated by the high level of genetic diversity of STR genotypes in the azole-containing compost. Our study confirms that azole resistance mutations continue to emerge in the environment and indicates compost containing azole residues as a possible hot spot. Better insight into the biology of environmental resistance selection is needed to retain the azole class for use in food production and treatment of *Aspergillus* diseases.

## IMPORTANCE

Composting of organic matter containing azole residues might be important for resistance development and subsequent spread of resistance mutations in *Aspergillus fumigatus*. In this article, we show the dominance of azole-resistant *A. fumigatus* in azole-exposed compost and the discovery of a new resistance mutation with clinical relevance. Furthermore, our study indicates that current fungicide application is not sustainable as new resistance mutations continue to emerge, thereby threatening the use of triazoles in medicine. We provide evidence that the sexual part of the fungal life cycle may play a role in the emergence of resistance mutations because under laboratory conditions, we reconstructed the resistance mutation through sexual crossing of two azole-resistant *A. fumigatus* isolates derived from the same compost heap. Understanding the mechanisms of resistance selection in the environment is needed to design strategies against the accumulation of resistance mutations in order to retain the azole class for crop protection and treatment of *Aspergillus* diseases.

## INTRODUCTION

**FIG 1:**
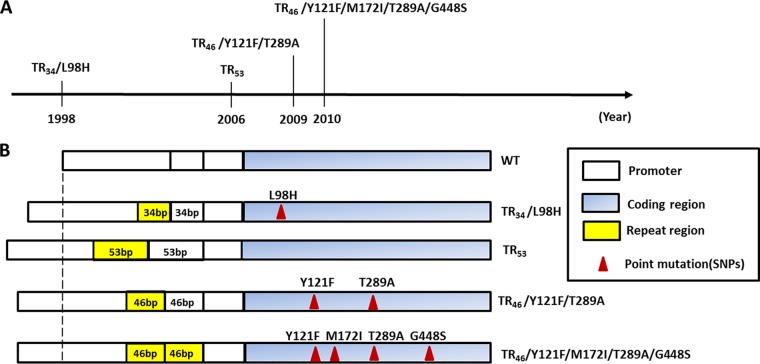
(A) Year in which for the first time since 1998 each of the different azole-resistant *A. fumigatus* isolates with TR promoter mutations was observed in The Netherlands. (B) Genotype illustration of the azole-resistance mutations in the *cyp51A* gene (promoter and coding region).

During the last decade, azole resistance has increasingly been reported in *Aspergillus fumigatus*, and it is now a global public health concern (1–11). Triazoles are the cornerstone of treatment of aspergillosis, and high mortality rates have been reported in patients with azole-resistant invasive aspergillosis (12–17). Azole resistance may emerge during azole therapy for patients; however, the extensive use of azole compounds in the environment is thought to be the major driver of resistance selection in *A. fumigatus* (18–20). The broad application of azoles for crop protection, material preservation, and clinical use has caused multiple resistance mutations to emerge. Although different azole compounds are used in the environment compared to those in medical applications, similarities in triazole molecule structure may explain the occurrence of strains that are cross-resistant to triazoles that are used for these different applications (19, 21). The majority of observed resistance mutations are caused by alterations in the *cyp51A* gene encoding the target protein sterol 14α-demethylase. Typically azole-resistant mutants have a combination of a tandem repeat (TR) in the promoter and single nucleotide polymorphisms (SNPs) in the coding sequence of this gene, such as TR_34_/L98H and TR_46_/Y121F/T289A (1, 10, 22–25). Figure 1 shows the history of discovery of promoter and coding region changes in the *cyp51A* gene of *A. fumigatus* in The Netherlands. These TR variants confer different azole resistance phenotypes, but most result in a pan-azole-resistant profile (26–32). The continued emergence of new resistance mutations can only be overcome if the critical steps in resistance development and selection are identified and understood.

Compost (decaying plant waste material) is believed to be an important biological niche for *A. fumigatus*, with high densities of conidiospores, where azole residues from agricultural waste may accumulate (33–35). Whether *A. fumigatus* is able to complete the whole life cycle in this harsh and competitive compost environment remains unclear. Use of variations in ways of reproduction exploiting various parts of the life cycle may be essential for resistance development, and such knowledge is thus critical for understanding potential hot spots of development, maintenance, and spread of azole resistance in *A. fumigatus*. We have previously shown that during azole exposure, resistance selection is enhanced when *A. fumigatus* reproduces asexually compared with nonsporulating controls (36). Beneficial spontaneous mutations produced during asexual reproduction were selected and accumulated over time. However, *A. fumigatus* can benefit from other reproduction modes, such as sexual or even parasexual reproduction, in order to create genetic variation (37). In the last decade, there has been accumulating evidence for cryptic sex in this fungus from population genetics studies, genome analysis, and the demonstration of a sexual cycle under laboratory conditions (38–44). However, direct observations or sampling of sexual structures in nature have not been reported, and the implication for resistance development is unclear.

In fungi, sexual reproduction can enhance adaptation to changing or new environments (45, 46) as sexual progeny have larger genetic variation compared with asexual populations. Greater genetic diversity is achieved through meiosis, by crossing over and chromosome reassortment. Genotyping studies of *A. fumigatus* show high diversity (39, 47–51), suggesting sexual reproduction in nature. In addition, both mating types are found in approximately equal proportions in such natural settings, as expected from balancing selection and Mendelian segregation in sexual crosses. Therefore, the sexual cycle, in addition to the asexual cycle, might play an important role in the ability of the fungus to adapt to the azole environment, and compost may provide the right conditions for sexual reproduction.

In this study, we investigated the azole phenotypes and genotypes of *A. fumigatus* in its natural habitat, the compost heap, but with contrasting azole exposures. One compost heap originated from organic waste with known azole exposure, while the other was azole free. Our hypothesis was that conditions that allow *A. fumigatus* to reproduce in the presence of azole pressure would select for azole-resistant *A. fumigatus* genotypes, whereas azole-sensitive *A. fumigatus* strains would be most frequent under azole-free conditions. In both settings, we searched for evidence of underlying reproduction modes, i.e., sexual versus asexual. In our opinion, insight into the dynamics of azole resistance selection of *A. fumigatus* in its natural habitat is essential to design strategies to prevent or reduce azole resistance development in the environment and ultimately help to manage the problem of azole-resistant *Aspergillus* diseases in patients.

## RESULTS

### Analysis of fungicide residues from the azole-free compost and azole-containing compost.

In the azole-free compost heap (Droevendaal, Wageningen, The Netherlands), no fungicide residues were detected (Table 1), while in the azole-containing compost from Hillegom, four fungicides were detected, three of which (prochloraz, prothioconazole, and tebuconazole) have been shown to cause cross-resistance to medical azoles (19) (Table 1). These fungicides are used in crop treatments—for instance for both spraying and immersion of bulbs prior to cultivation. In addition, in the azole-containing compost sample, the insecticide imidacloprid and another fungicide, boscalid, were detected. These pesticides are also used in conventional bulb cultivation in The Netherlands.

**TABLE 1:** Fungicides detected from the azole-free compost samples W5 and W6 and azole-containing compost samples H1 and H4

Sample(s)	Detected fungicide	Fungicide concn (mg/kg)
W5, W6	None	
H1	Prothioconazole-desthio	0.250
	Prochloraz	0.020
	Imidacloprid	0.013
	Boscalid	0.026
H4	Tebuconazole	0.055
	Prothioconazole-desthio	0.068

### Isolation of *A. fumigatus* from azole-containing and azole-free compost before and after heat shock.

**FIG 2:**
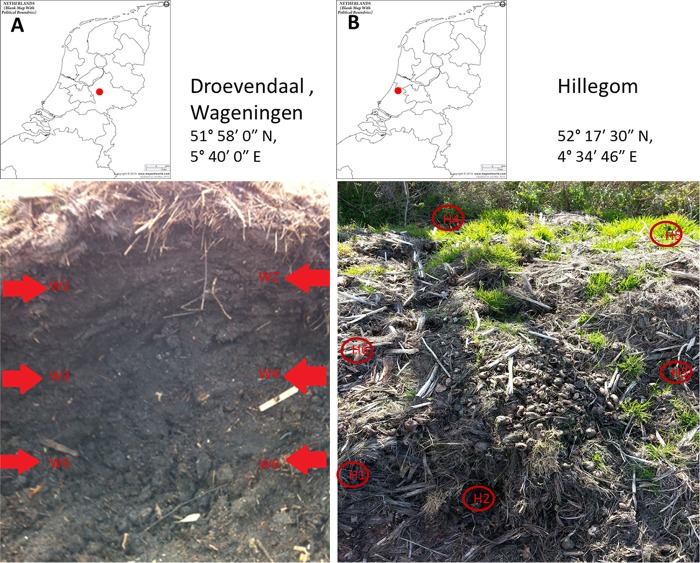
Locations and positions of samples taken from azole-free compost in Wageningen (W1 to W6 [30 cm apart from top to bottom]) and azole-containing compost in Hillegom (H1 to H6).

**FIG 3:**
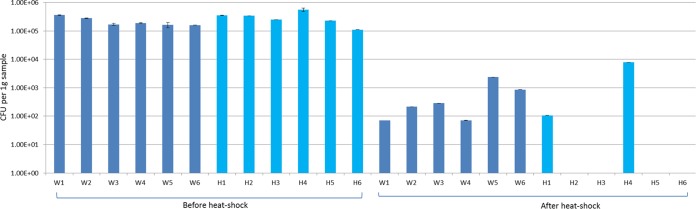
*A. fumigatus* CFU density (CFU per gram of compost) before and after heat shock in samples taken at different positions in an azole-free compost heap (W1 to W6 [dark blue bars]) and an azole-containing compost heap (H1 to H6 [light blue bars]).

Before and after heat shock of each of the six samples from the two sample locations (Fig. 2), colonies with macroscopic and microscopic characteristics of *A. fumigatus* were recovered. From each sample, 20 randomly picked colonies were confirmed as *A. fumigatus* based on sequence analysis and their ability to grow at 48°C. Before heat shock, each sample contained more than 10^5^ CFU. After heat shock, all six samples from the nonazole compost heap (W1 to W6) and two of the six samples from the azole compost heap (H1 and H4) yielded *A. fumigatus* isolates ranging from 71 to ~8,000 CFU per g of compost (Fig. 3).

### Azole resistance phenotypes and genotypes.

Azole-resistant *A. fumigatus* was found in both compost heaps, although the proportion of resistance was different, with 91% (41/45 colonies) resistance in the azole-containing compost compared to only 2% (1/50) in the azole-free compost. The genotypic variation in the *cyp51A* gene differed in the azole-free compost (98% wild type [WT], 2% TR_34_/L98H) and the azole-containing compost (9% WT, 80% TR_46_Y121F/T289A, and 2% TR_46_Y121F/M172I/T289A/G448S) (Tables 2 and 3). Moreover, in 9% of the phenotypic azole-resistant isolates, a new mutation was identified, consisting of a triple repeat of 46 bp of the promoter region that is present in duplicate in TR_46_ (TR_46_^3^) combined with four mutations in the *cyp51A* gene, namely Y121F, M172I, T289A, and G448S.

**TABLE 2:** Genetic variation in azole-containing compost sample H4 from plant material waste in Hillegom, The Netherlands^a^

AZN_NMR no.	Sample	STR3			STR4			Cyp51A	Cyp51A SNP	Cyp51A SNP	Cyp51A SNP	Cyp51A	Mating type	Genotype × frequency
		A	B	C	A	B	C							
V181-07	H1	33	10	23	6	13	8	TR_46_	Y121F	None	T289A	None	MAT1-2	1 × 3
V181-08	H2	44	8	61	6	12	8	TR_46_	Y121F	None	T289A	None	MAT1-2	2 × 1
V181-09	H3	44	8	7	10	9	7	TR_46_	Y121F	None	T289A	None	MAT1-2	3 × 11
V181-11	H5	42	8	61	7	10	8	WT	None	None	None	None	MAT1-1	4 × 2
V181-12	H6	35	9	27	8	10	8	WT	None	None	None	None	MAT1-1	5 × 1
V181-13	H7	44	8	12	10	8	18	TR_46_	Y121F	None	T289A	None	MAT1-2	6 × 1
V181-14	H8	35	11	16	8	7	6	WT	None	None	NONE	None	MAT1-1	7 × 1
V181-15	H9	42	9	62	10	8	7	TR_46_	Y121F	None	T289A	None	MAT1-2	8 × 2
V181-16	H10	46	8	7	10	8	7	TR_46_	Y121F	None	T289A	None	MAT1-1	9 × 2
V181-19	H13	43	8	11	10	8	18	TR_46_	Y121F	None	T289A	None	MAT1-2	10 × 1
V181-20	H14	33	10	23	6	10	13	TR_46_	Y121F	None	T289A	None	MAT1-2	11 × 1
V181-23	H17	31	8	21	6	12	8	TR_46_	Y121F	None	T289A	None	MAT1-2	12 × 2
V181-32	H26	44	8	12	2	8	6	TR_46_	Y121F	None	T289A	None	MAT1-2	13 × 2
V181-34	H28	55	8	7	6	8	8	TR_46_	Y121F	None	T289A	None	MAT1-1	14 × 1
V181-35	H29	44	8	7	12	9	9	TR_46_	Y121F	M172I	T289A	G448S	MAT1-2	15 × 1
V181-36	H30	26	8	12	6	9	7	TR_46_^3^	Y121F	M172I	T289A	G448S	MAT1-1	16 × 2
V181-38	H32	26	7	12	6	8	7	TR_46_	Y121F	None	T289A	None	MAT1-1	17 × 1
V181-39	H33	41	8	7	10	8	8	TR_46_^3^	Y121F	M172I	T289A	G448S	MAT1-2	18 × 1
V181-40	H34	25	18	24	8	10	9	TR_46_	Y121F	None	T289A	None	MAT1-1	19 × 1
V181-43	H37	22	4	13	6	9	9	TR_46_^3^	Y121F	M172I	T289A	G448S	MAT1-1	20 × 1
V181-44	H38	31	8	23	6	13	8	TR_46_	Y121F	None	T289A	None	MAT1-2	21 × 1
V181-46	H40	26	8	21	11	8	7	TR_46_	Y121F	None	T289A	None	MAT1-1	22 × 1
V181-48	H42	44	8	7	9	9	9	TR_46_	Y121F	None	T289A	None	MAT1-2	23 × 1
V181-49	H43	24	6	20	12	8	7	TR_46_	Y121F	None	T289A	None	MAT1-1	24 × 1
V181-50	H44	44	8	12	10	8	7	TR_46_	Y121F	None	T289A	None	MAT1-2	25 × 1
V181-51	H45	44	8	17	2	3	7	TR_46_	Y121F	None	T289A	None	MAT1-2	26 × 1

a STR3 A, B, and C and STR4 A, B, and C are six microsatellite markers. The genotype counting is based on the difference between the STR3 A, B, and C and STR4 A, B, and C markers (differences higher than 2 repeats), CYP51 promoter, and point mutation in the coding region and mating type. The genotype classification is based on whether the number of STR repeat differences is higher than 2 repeats.

**TABLE 3:** Genetic variation in azole-free compost sample W5 from an organic plant waste compost heap in Wageningen, The Netherlands^a^

AZN_NMR no.	Sample	STR3			STR4			Cyp51A	Cyp51A	Mating type	Genotype × frequency
		A	B	C	A	B	C				
V182-18	W1	34	9	8	7	8	31	WT	WT	MAT1-2	1 × 1
V182-19	W2	38	8	12	8	10	20	WT	WT	MAT1-1	2 × 1
V182-20	W3	30	8	11	8	9	20	WT	WT	MAT1-2	3 × 4
V182-22	W5	38	10	46	10	8	8	WT	WT	MAT1-1	4 × 1
V182-23	W6	29	7	11	9	9	18	WT	WT	MAT1-1	5 × 1
V182-24	W7	26	10	21	8	7	5	WT	WT	MAT1-2	6 × 1
V182-25	W8	30	8	11	8	9	20	WT	WT	MAT1-2	7 × 1
V182-27	W10	25	8	8	6	9	8	WT	WT	MAT1-2	8 × 1
V182-28	W11	13	18	8	7	26	5	WT	WT	MAT1-2	9 × 1
V182-29	W12	30	8	11	5	9	20	WT	WT	MAT1-2	10 × 1
V182-30	W13	9	8	8	8	8	6	WT	WT	MAT1-2	11 × 1
V182-31	W14	30	8	8	7	9	19	WT	WT	MAT1-1	12 × 2
V182-32	W15	13	8	10	8	8	3	WT	WT	MAT1-1	13 × 1
V182-33	W16	30	8	8	5	9	19	WT	WT	MAT1-1	14 × 1
V182-34	W17	27	8	30	7	6	5	WT	WT	MAT1-2	15 × 1
V182-35	W18	27	8	26	10	8	5	WT	WT	MAT1-2	16 × 4
V182-36	W19	24	8	8	8	11	33	WT	WT	MAT1-1	17 × 2
V182-37	W20	50	8	8	8	9	25	WT	WT	MAT1-2	18 × 1
V182-38	W21	10	8	8	5	7	18	WT	WT	MAT1-1	19 × 1
V182-39	W22	30	8	11	5	7	18	WT	WT	MAT1-2	20 × 1
V182-40	W23	36	10	30	22	9	8	WT	WT	MAT1-1	21 × 1
V182-42	W25	50	8	8	10	8	5	WT	WT	MAT1-1	22 × 1
V182-43	W26	8	7	11	7	8	10	WT	WT	MAT1-1	23 × 1
V182-45	W28	30	8	11	8	9	21	WT	WT	MAT1-2	24 × 1
V182-46	W29	25	8	21	14	8	5	WT	WT	MAT1-2	25 × 1
V182-51	W34	28	10	8	8	8	8	WT	WT	MAT1-1	26 × 1
V182-52	W35	29	16	8	10	26	5	WT	WT	MAT1-2	27 × 1
V182-54	W37	38	8	12	5	10	21	WT	WT	MAT1-1	28 × 1
V182-55	W38	32	8	8	7	9	19	WT	WT	MAT1-2	29 × 3
V182-57	W40	28	9	12	8	6	11	TR_34_	L98H	MAT1-1	30 × 1
V182-58	W41	36	8	9	7	9	8	WT	WT	MAT1-2	31 × 1
V182-59	W42	45	9	10	10	8	10	WT	WT	MAT1-1	32 × 1
V182-60	W43	37	8	20	6	7	5	WT	WT	MAT1-1	33 × 1
V182-61	W44	29	8	11	8	9	27	WT	WT	MAT1-2	34 × 1
V182-63	W46	34	8	8	5	9	8	WT	WT	MAT1-2	35 × 1
V182-64	W47	37	22	21	6	8	5	WT	WT	MAT1-2	36 × 1
V182-65	W48	39	8	8	8	9	21	WT	WT	MAT1-2	37 × 1
V182-66	W49	15	8	20	5	7	5	WT	WT	MAT1-2	38 × 1
V182-67	W50	26	10	8	31	29	6	WT	WT	MAT1-2	39 × 1

a STR3 A, B, and C and STR4 A, B, and C are six microsatellite markers. The genotype counting is based on the difference between STR3 A, B, and C and STR4 A, B, and C (differences higher than 2 repeats), CYP51 promoter, and point mutation in the coding region and mating type. The genotype classification is based on whether the number of STR repeat differences is higher than 2 repeats.

The ratio of MAT1-1 to MAT1-2 among the different genotypes was not significantly different from the expected 1:1 ratio (11:15 and 15:21 for the azole-containing and azole-free composts, respectively; chi-square values of *P* = 0.432 and *P* = 0.317). Ample genetic variation was present in both compost heaps based on six microsatellite markers; 26 unique genotypes from the azole-containing heap and 39 from the azole-free heap were identified (Tables 2 and 3).

### Susceptibility testing and gene expression of TR_34_, TR_46_, and TR_46_^3.^

**FIG 4:**
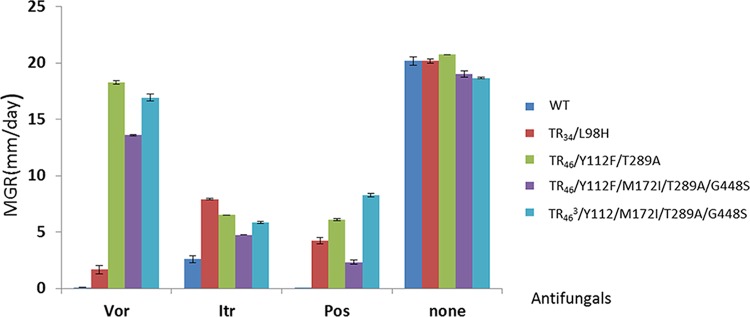
Azole-resistance assay based on MGR of the TR_34_, TR_46_, and TR_46_^3^ variants against voriconazole (Vor), itraconazole (Itr), and posaconazole (Pos). MEA was supplemented with 2 mg/liter of Vor and Itr, respectively, with Pos at 0.5 mg/liter. None, without any azoles. Error bars indicate the standard error of the mean (SEM). The MGR was determined by averaging the colony diameters (in millimeters) as measured in two randomly chosen perpendicular directions.

The mycelial growth rate (MGR) of the TR variants TR_34_, TR_46_, and TR_46_^3^ isolated from the two compost heaps is shown in Fig. 4. TR_46_^3^/Y121F/M172I/T289A/G448S exerted the fastest mycelial growth rate on medium supplemented with posaconazole, compared with TR_34_/L98H and TR_46_/Y121F/T289A (analysis of variance [ANOVA], *F*_4, 10_ = 369.674; *P* < 0.01) (Fig. 4). In addition, to compare the susceptibility of TR_46_^3^ with those of TR_34_ and TR_46_, the high resistance of TR_46_^3^/Y121F/M172I/T289A/G448S was confirmed by MIC testing, displaying a pan-triazole-resistant phenotype to posaconazole, itraconazole, and voriconazole (Table 4), indicating no *in vitro* activity of itraconazole and voriconazole (MIC, >16 mg/liter) (26).

**TABLE 4:** *In vitro* activities of itraconazole, voriconazole, and posaconazole against *A. fumigatus* isolates harboring TR_46_^3^ compared with published resistance profiles of TR_34_, and TR_46_

Isolate type	MIC range (mg/liter) for^a^:			Source or reference
	Itr	Vor	Pos	
TR_34_/L98H	>16	4–8	0.25–0.5	5
TR_46_/Y121F/T289A	4/16	>16	0.25–2	21
TR_46_^3^/Y121F/M172I/T289A/G448S	>16	>16	1	This study^b^

a Itr, itraconazole; VOR, voriconazole; POS, posaconazole.

b Two isolates from compost plus 3 clinical isolates.

**FIG 5:**
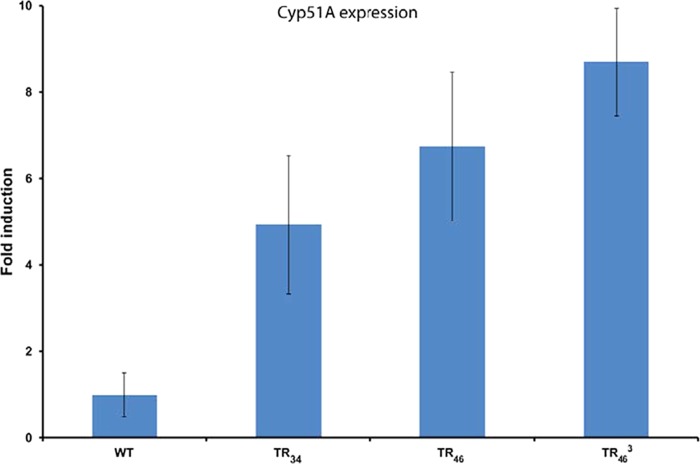
Gene expression levels of c*yp51A* in TR variants (WT TR_34_, TR_46_, and TR_46_^3^). Bars show averages from nine replicates, and error bars indicate the standard error of the mean (SEM).

Expression levels assayed from Cyp51A/actin mRNA ratios showed that even in the absence of azoles, *cyp51A* gene expression levels in the TR_46_^3^/Y121F/M172I/T289A/G448S strain were significantly higher than those in the WT by ANOVA (*F*_3, 7_ = 38.279, and *P* < 0.001) and post hoc least significant difference (LSD) test (*P* < 0.01 for TR_46_^3^/Y121F/M172I/T289A/G448S versus TR_34_/L98H and *P* < 0.05 for TR_46_^3^/Y121F/M172I/T289A/G448S versus TR_46_/Y121F/T289A), which suggests that the repeats in the promoter region are able to generate more *cyp51A* transcript and likely CYP51 protein to cope with azole stress and therefore contribute to a resistant phenotype (Fig. 5).

### Analysis of clinical isolates from the Dutch national surveillance program.

The *A. fumigatus* culture collection of the Dutch national *Aspergillus* resistance surveillance was used to determine whether TR_46_^3^/Y121F/M172I/T289A/G448S was present in clinical isolates. All clinical *A. fumigatus* isolates collected between 2012 and 2015 that harbored a Y121F mutation were investigated for the presence of TR_46_^3^ by PCR. Among 98 Y121F-harboring isolates, 3 were identified with TR_46_^3^. In addition, one isolate was found with four TRs (TR_46_^4^) in combination with the four mutations in the *cyp51A* gene: TR_46_^4^/Y121F/M172I/T289A/G448S.

One TR_46_^3^/Y121F/M172I/T289A/G448S isolate was recovered from a sputum sample from a 34-year-old female in 2013 in the western part of The Netherlands. The patient was hospitalized for treatment of synovial cell sarcoma with pleural metastasis. Computed tomography (CT) of the lungs showed no evidence for *Aspergillus* disease. A second patient was admitted to a hospital in the northwest part of The Netherlands in 2015. The patient was treated for asthma as an outpatient because of shortness of breath. Again, CT of the chest showed no evidence for *Aspergillus* disease.

A third patient was identified through an *A. fumigatus* isolate that was sent in for MIC testing. The isolate was cultured in 2012 from a 43-year-old patient with chronic obstructive pulmonary disease (COPD) admitted to a hospital in the eastern part of The Netherlands. The patient was admitted with a community-acquired pneumonia and received therapy with posaconazole in addition to antibacterial therapy. Because sputum cultures remained positive after 2 weeks of posaconazole therapy, the strain was sent for MIC testing. However, a definite diagnosis of invasive pulmonary aspergillosis could not be made.

The TR_46_^4^ isolate was cultured in 2012 from the right ear of a 34-year-old male who was treated in the northern part of the country for cholesteatoma. The fungus may have contributed to the chronic infection, but there was no evidence for invasive disease.

### The generation of TR_46_^3^ via sexual reproduction of two TR_46_ strains from the azole-containing compost.

To obtain proof of principle that sexual reproduction could cause TR mutations, we performed a sexual cross between a pair of strains from the same azole-containing compost sample with the TR_46_/Y121F/T289A mutation but of opposite mating types (isolates H40 [MAT1-1/TR_46_/Y121F/T289A] and H35 [MAT1-2/TR_46_/Y121F/T289A]) (38, 44). After 4 months, cleistothecia were harvested, and 10^5^ ascospores were heat shocked and plated on malt extract agar (MEA) with a high concentration of posaconazole (2 mg/liter), which showed the largest difference in MGR between TR_46_ (nearly no growth) and TR_46_^3^ (with growth). Five hundred colonies that grew well in the presence of posaconazole were selected for sequence analysis of the *cyp51A* gene. Of these, 499 isolates were TR_46_/Y121F/T289A, but one colony contained the novel TR_46_^3^/Y121F/T289A mutation. This shows that TR_46_^3^ can indeed arise from a sexual cross between isolates with TR_46_/Y121F/T289A under laboratory conditions.

## DISCUSSION

### Discovery of novel azole resistance mutations and the hot spot for resistant *A. fumigatus.*

In this study, we observed that azole-containing compost harbored predominantly azole-resistant *A. fumigatus*, whereas in azole-free compost almost exclusively wild-type *A. fumigatus* was found. Our study suggests that azole exposure to *A. fumigatus* in its natural habitat at least sustains the presence of azole resistance. This, and similar habitats, could act as a “hot spot”—i.e., providing a source of azole-resistant *A. fumigatus* from which airborne conidia may migrate and cause disease in susceptible hosts, contribute to further azole resistance development, or both.

We identified a new resistance mutation (TR_46_^3^/Y121F/M172I/T289A/G448S) in the azole-exposed compost. Analysis of clinical isolates that had been collected in the national surveillance program of The Netherlands showed that since 2012, each year at least one patient harbored an isolate with this new resistance mutation. Little is known about the generation mechanism and migration rate of resistant *A. fumigatus*, but the observation that the TR_34_/L98H variant is now the most frequently reported mutation globally (1–5, 7–10, 52, 53) suggests that spores can rapidly spread from a single source and over long distances. The TR_46_/Y121F/T289A mutation also showed rapid migration, as after its discovery in December 2009, the mutation was recovered from patients from six different hospitals in The Netherlands within 1 year (26). In line with these observations, the TR_46_^3^/Y121F/M172I/T289A/G448S mutation was recovered from unrelated patients in three different hospitals in The Netherlands over a 3-year period, indicating that the resistance mutation might have migrated across the country, but at a slower pace compared to the other mutations. Although the mutation was found to only colonize patients, we believe that isolates harboring TR_46_^3^/Y121F/M172I/T289A/G448S may ultimately cause invasive infections—potentially after accumulation of additional mutations allowing adaptation to the human host—as the resistance trait has probably survived in the environment for several years, in competition with other azole-resistant or wild-type isolates, indicating comparable fitness. This would imply that the cost of resistance of carrying TR_46_^3^ is limited and/or that compensatory mutations easily emerge since otherwise selection would act against this mutation. In addition to TR_46_^3^, a clinical azole-resistant isolate with four copies of TR_46_ was found. Although this genotype was not recovered from the environment, we believe that given the similarity of the resistance mutations to other TR_46_ variants, it originated via similar mechanisms to the other TR mutations, which included exposure to azole fungicides. These observations confirm our concern that new azole resistance mutations will continue to emerge in the environment if we persevere with our current practices of azole fungicide use. The azole-containing habitat may serve as an evolutionary incubator with increased selective pressure on recombination that benefits the fungus through increased fitness or increased resistance, thus facilitating its survival. With increasing azole resistance frequencies and greater diversity in resistance mutations, the consequence will be that the clinical role of medical triazoles for the management of *Aspergillus* diseases will be marginalized. As the arsenal of available drugs to treat *Aspergillus* diseases is already very limited, excess mortality due to azole resistance can be expected (54). Indeed, case studies show high mortality rates in patients with azole-resistant invasive aspergillosis (12, 16, 17, 55). Environmental resistance is especially difficult to manage as it is found in any *Aspergillus* disease and may occur in any patient, even those without previous azole therapy (54). Recently, mixed infections due to azole-susceptible and azole-resistant *A. fumigatus* strains were reported, which further complicates timely diagnosis and successful therapy (56, 57).

Our findings therefore underscore the urgent need to further investigate the evolution of *A. fumigatus* resistance in the environment as this will provide leads that enable us to prevent new mutations from emerging. In this way, investigation of the evolution of resistance may allow earlier detection and earlier intervention, especially in modification of patient therapy.

### The role of sexual reproduction in the emergence of TR_46_^3^/Y121F/M172I/T289A/G448S.

**FIG 6:**
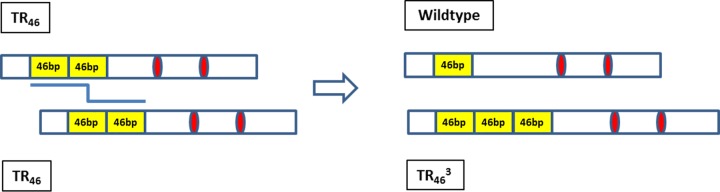
A possible scenario indicating how unequal crossing over in a sexual cross between two strains with a double repeat TR_46_ can result in a rare meiotic recombinant with the triple repeat TR_46_^3^. Yellow boxes represent the tandem repeats in the promoter region of *cyp51A* gene, and red ovals represent point mutations in the coding region of the *cyp51A* gene. The bent blue line indicates unequal crossover.

Theoretically, the isolates with TR_46_^3^ mutations could have originated through both asexual and sexual reproduction. However, heat-shock treatment reduced the number of *A. fumigatus* CFU dramatically (Fig. 3), suggesting that most, if not all, conidiospores were killed and leaving only the heat-resistant ascospores in the compost sample. Furthermore, a high genetic diversity was found in the heat-shocked compost samples, as shown in Tables 2 and 3 for microsatellite loci and mating-type genes, which is indicative of sampling unique sexual spores rather than clonal conidiospores. Importantly, within the population the high microsatellite diversity and equal mating-type distribution are both consistent with sexual reproduction. Through sexual recombination, a multitude of combinations of microsatellite markers and mating-type loci can be generated, whereas balancing selection for the mating-type locus and Mendelian segregation accounts for equal distribution of the two mating types. Clonal expansion by asexual reproduction on the other hand would result in a population with low genetic variation and an unbalanced mating-type ratio. Here, we found TR_46_^3^ isolates of either mating type and of different microsatellite types. Therefore, we expected that the novel TR_46_^3^ genotype originated from a sexual cross; we tested progeny from a cross between two TR_46_ strains and were indeed able to recover this genotype. One mechanism that may underpin this new resistance genotype may have been unequal meiotic crossing over, as illustrated in Fig. 6 (58). The mispairing in the repeat region of 46 bp during the meiotic process may lead to even longer repeats in the promoter. As expected, the TR_46_^3^ strain obtained from the cross contained only the two mutations Y121F and T289A present in the parental TR_46_ strains. The two additional mutations M172I and G448S, which are found in the natural TR_46_^3^ isolates, must then have come from parental strains with TR_46_, of which one or both have all four mutations. Thus, this important observation suggests the potential occurrence of sex in the compost.

In addition to these observations that support the notion of sexual reproduction, it is important to note that compost heaps provide favorable conditions for sex. Several key factors for sexual reproduction that have been identified under laboratory conditions seem to be present in compost (38). Compost contains multiorganic resources, with inside the heap a warm, dark, low-O_2_, and high-CO_2_ environment as a result of biological metabolic activity. A dynamic composting process with temperature gradients (20 to 70°C) and gas changes might therefore stimulate sexual reproduction of *A. fumigatus* (45, 59). The extent to which these favorable conditions for sex are present in the compost may differ for specific compost samples, which may explain the difference in number of ascospores between the two compost samples after heat shock (Fig. 3).

However, we are aware that the presence of sexual reproduction was not definitively proven in our study. The effectiveness of the heat shock treatment was proven with a suspension containing ascospores, but not asexual conidiospores, that were able to survive a heat shock of 70°C for 1 h (38, 60). However, whether the same effectiveness can be reached in a complex matrix such as compost has not yet been determined. We found that although indeed the ascospores were heat resistant in compost, not all of the 10^5^ conidiospores were killed after 1 h at 70°C (J. Zhang, personal observation). A previous study has also shown that a population of conidiospores may not completely be killed by heat shock, depending on many factors, such as the freshness and concentration of conidiospores, the type of strain, and the pH (61). Therefore, we cannot definitively conclude that all colonies derived after heat shock of compost material have originated from ascospores. However, the significant reduction in the number of CFU of *A. fumigatus* after heat shock indicates that most if not all, conidiospores were killed. Furthermore, the observed genotype distribution among isolates obtained from heat-treated compost (Table 2) provides strong evidence that these isolates do originate from recombination through sexual reproduction. Direct light microscopic observation of ascospores in compost samples failed as they could not be clearly recognized, even after addition of ascospores (unpublished observations).

### Conclusions and future outlook.

Our study shows that azole-exposed compost can serve as a hot spot as almost exclusively azole-resistant phenotypes were found there. In addition to known resistance mutations, we found two new mutations and evidence that these might evolve through sexual reproduction. Our data clearly indicate that the full life cycle of *A. fumigatus* needs to be taken into account to explain the emergence of azole resistance. The continued emergence of azole resistance mutations warrants research into the mechanisms of resistance selection in the environment. Insights into key factors involved in the selection or spread of resistance in *A. fumigatus* will help to develop strategies that prevent resistance selection. Only then can this important class be rescued for use for crop protection as well as treatment of *Aspergillus* diseases.

## MATERIALS AND METHODS

### Sampling and screening of *A. fumigatus* in azole-free and azole-containing compost.

Six samples were collected from each of two compost heaps: one originating from waste containing azoles (Hillegom, The Netherlands) and one free of azoles (Wageningen, The Netherlands) (Fig. 2). To document the presence of azole fungicides, two samples from each compost heap were analyzed for fungicide residues using gas chromatography-tandem mass spectrometry (GC-MS/MS) and liquid chromatography-tandem mass spectrometry (LC-MS/MS) by Eurofins lab—Zeeuws Vlaanderen (http://www.labzvl.nl/en).

Samples of 1 g compost were added to 10 ml sterile saline with 0.05% Tween and screened for the presence of *A. fumigatus* by plating 50-µl samples on malt extract agar (MEA [36]) supplemented with two antibiotics (10 µg/ml streptomycin and 15 µg/ml tetracycline [Sigma-Aldrich, Germany]) and incubated at 37°C. Ten randomly selected colonies from each compost heap, which all showed *Aspergillus* morphology, were identified as *A. fumigatus* genetically by sequencing of the β-tubulin and carboxypeptidase-5 genes (38) and phenotypically by their capacity to grow at 48°C. The presence of *A. fumigatus* ascospores was investigated by subjecting the sample to heat shock (1 h at 70°C) before plating. This heat shock procedure has been shown to eliminate and greatly reduce *A. fumigatus* conidiospores (resulting from asexual reproduction), but not ascospores (resulting from sexual reproduction) (38, 41). As a consequence, any colony growing after heat shock is likely to have originated from a sexual spore. After 2 days, the colonies were counted and the survival rate was established.

### Azole resistance phenotypes and genotypes of *A. fumigatus* cultures from compost.

As we were focused in this study on the sexual route of resistance development, we randomly picked 50 presumed ascospore-derived isolates after heat shock from a W5 (azole-free) compost sample and an H4 (azole-containing) compost sample with the highest *A. fumigatus* count. These isolates were genotyped for six microsatellite markers (STR3 A, B, and C and STR4 A, B, and C) and the *cyp51A* gene and its promoter region (62–64). The mating type was determined by sequencing the mating-type gene (40).

### Susceptibility testing and *cyp51A* gene expression of TR variants (TR_34_, TR_46_, and TR_46_^3^) found from compost.

Since we previously found that the level of resistance based on mycelial growth rate (MGR) is highly correlated with the results from the MIC test, here we used the more straightforward and reproducible MGR measurement as described before (36). The MGR of TR variants (TR_34_, TR_46_, and TR_46_^3^) that were isolated from the compost heaps was assayed under the condition of exposure to three medical triazoles (itraconazole, voriconazole, and posaconazole). In addition, to compare the susceptibility of TR_46_^3^ with those of TR_34_ and TR_46_, the high resistance of TR_46_^3^ was confirmed by MIC testing, using a broth microdilution method according to EUCAST protocol E.DEF 60 9.2 (36, 65). The *cyp51A* expression was analyzed from duplicate cultures of three different strains per TR variant. Expression levels were calculated from Cyp51A/actin mRNA ratios and normalized for wild-type (WT) expression levels (25).

### Clinical implications of environmental azole resistance mutations.

In five Dutch University Medical Centres, clinical *A. fumigatus* isolates are routinely screened for azole-resistant phenotype using agar supplemented with medical triazoles. Isolates that grow in the presence of azoles were sent to the National Mycology Reference Laboratory at Radboud University Medical Centre for MIC testing and genotypic characterization. Screening for triazole resistance mutations was performed using the Y121F mutation for the TR_46_/Y121F/T289A genotype and L98H for the TR_34_/L98H genotype, respectively (66). These two mutations have been shown to account for over 80% of clinical triazole resistance. If none of these mutations were found in the resistant isolates, the full *cyp51A* gene was sequenced. In this study, for all L98H- and Y121F-positive isolates collected between 2012 and 2015, the accompanying TR was determined by PCR analysis (67), and for selected isolates, relevant clinical information was retrieved.

### Sexual cross between two TR_46_ strains of opposite mating types from the same azole-containing compost.

As our results indicated a possible involvement of sexual reproduction in the development of the new resistance mutation, we investigated the possibility that TR_46_^3^ mutations arise through sexual reproduction, so a pair of strains from the same azole-containing compost sample with the TR_46_ resistance mutation and of the opposite mating type (isolates H40 [MAT1-1/TR_46_/Y121F/T289A] and H35 [MAT1-2/TR_46_/Y121F/T289A]) were used for a sexual cross following the protocol of O’Gorman et al. (38). After 4 months, cleistothecia were harvested, and 10^5^ ascospores were heat shocked and plated on MEA with posaconazole (2 mg/liter) for selection of TR_46_^3^.

### Statistical analysis.

We tested the significance of differences in MGR and gene expression of the *cyp51A* gene of the different TR variants using a one-way ANOVA with number of repeats as a nominal factor. Pairwise differences between variants were tested post hoc using Fisher’s least significant difference (LSD). Whether the mating-type distribution in the two composts was a deviation from a 1:1 ratio was tested using a chi-square test.
